# Icariin Improves Age-Related Testicular Dysfunction by Alleviating Sertoli Cell Injury *via* Upregulation of the ER*α*/Nrf2-Signaling Pathway

**DOI:** 10.3389/fphar.2020.00677

**Published:** 2020-05-12

**Authors:** Haixia Zhao, Xu You, Qian Chen, Siqi Yang, Qiongyan Ma, Yumin He, Chaoqi Liu, Yaoyan Dun, Jie Wu, Changcheng Zhang, Ding Yuan

**Affiliations:** ^1^College of Medical Science, China Three Gorges University, Yichang, China; ^2^Third-Grade Pharmacological Laboratory on Chinese Medicine Approved by State Administration of Traditional Chinese Medicine, China Three Gorges University, Yichang, China; ^3^The Second People’s Hospital of Yichang, China Three Gorges University, Yichang, China; ^4^Material Analysis and Testing Center, China Three Gorges University, Yichang, China

**Keywords:** Sertoli cell, ER*α*/Nrf2, icariin, testis, aging

## Abstract

Sertoli cells play crucial roles in spermatogenesis and are impaired by aging. Icariin, a flavonoid from *Epimedium*, has been reported to exhibit anti-aging effects and improve testicular dysfunction in the clinical setting. However, whether icariin improves age-related degeneration of testicular function *via* protection from Sertoli cell injury remains unclear. In the present study, we evaluated the protective effect of icariin on Sertoli cell injury and explored the possible mechanism(s) *in vivo* and *in vitro*. Dietary administration of icariin for 4 months significantly ameliorated the age-related decline in testicular function by increasing testicular and epididymal weights and indices, sperm count and sperm viability, testicular testosterone and estradiol concentrations, and seminiferous tubule diameters and heights. In addition, icariin protected age-related Sertoli cells from injury as evidenced by an analysis of Sertoli cell number, ultrastructure, and function. Such changes were accompanied by upregulation of ER*α* and Nrf2 signaling in Sertoli cells. Parallel *in vitro* studies also demonstrated that icariin inhibited untoward effects on the TM4 mouse Sertoli cell line with concomitant upregulation of ER*α* and Nrf2 signaling. Conversely, ER*α* siRNA reversed icariin-mediated protection of Sertoli cell injury. Our data suggest that icariin effectively ameliorates age-related degeneration of testicular function by alleviating Sertoli cell injury *via* the ER*α*/Nrf2 signal-transduction pathway. Thus, mitigating Sertoli cell damage *via* the ER*α*/Nrf2 signaling pathway likely represents a promising strategy for the prevention of age-related testicular dysfunction.

## Introduction

The Sertoli cell is the key somatic cell in the testis that facilitates testis formation and spermatogenesis by producing various proteins, including glial cell-derived neurotrophic factor (GDNF), promyelocytic leukemia zinc finger (PLZF), bone morphogenetic protein 4 (BMP4), and stem cell factor (SCF) (França et al., 2016; Vogl et al., 2018). These factors promote spermatogonial stem cell (SSC) self-renewal and differentiation to ensure that spermatogonia develop into mature spermatozoa. Previous studies have suggested that Sertoli cells injury—including the loss of Sertoli cells’ number and function—is directly related to degeneration of testicular reproductive function with aging (Johnson et al., 1984; Anderson, 2000). Clinical studies have shown that abnormal changes in Sertoli cell morphology are observed with increasing age and that these changes are accompanied by a significant reduction in levels of proteins secreted by Sertoli cells, loss of germ cells, and increased germ cell apoptosis (Tenover et al., 1988; Jiang et al., 2014). Furthermore, the ultrastructure of Sertoli cells in the aging human testis showed that there are abundant lipid droplets, large cytoplasmic vacuoles filled with an amorphous material, numerous mitochondria that display tubular cristae, and loose endoplasmic reticula (Paniagua et al., 1985; Jiang et al., 2014). Thus, protecting Sertoli cells from injury during aging represents an effective strategy for age-related dysfunction of the testis.

Estrogen and estrogen receptors (including the classical estrogen receptors ER*α* and ER*β*, and G protein-coupled estrogen receptor 1 [GPER]) have been reported to be important for the proliferation and function of Sertoli cells (Lucas et al., 2008; Lucas et al., 2011). A previous study showed that ER*α* and ER*β* promoted Sertoli cell proliferation and that GPER—but not ER*α* and ER*β*—modulated gene expression involved with Sertoli cell apoptosis (Royer et al., 2012). Clinical studies have found that estradiol levels declined with aging in humans (Carreau et al., 2004) and that ER*α* expression diminished in Sertoli cells of men with obstructive and nonobstructive azoospermia (Han et al., 2009). Furthermore, estrogen-dependent ER*α* signaling is essential for germ cell viability, most likely through Sertoli cell functioning (Sinkevicius et al., 2009). Investigators have also recently found that the concentration of estrogen and the expression of ER*α* are also significantly decreased in the testis of naturally aging rats and mice (Banerjee et al., 2012; Clarke and Pearl, 2014). Conversely, exogenous estrogen treatment attenuated the age-related loss in ER*α* expression and sperm production in naturally aging rats, although ER*β* expression was not altered during aging or after treatment with estrogen (Clarke and Pearl, 2014). Therefore, estrogen and ER*α* might be important for Sertoli cell survival and function. However, whether estrogen and ER*α* exert protective effects with respect to Sertoli cell injury due to aging has not yet been elucidated.

The nuclear factor-E2-related factor 2 (Nrf2)-signaling pathway, a key cellular protective signaling pathway against reactive oxygen species (ROS) and chronic oxidative stress, has been frequently shown to be inactivated with aging and is hypothesized to be an appealing therapeutic target of aging and various age-related diseases, including age-related testicular dysfunction and age-related macular degeneration (Chapple et al., 2012; Salomon et al., 2013; Aydın et al., 2015; Zhu et al., 2015). Several lines of evidence suggest that estrogen *via* its receptors induces rapid activation of Nrf2 in different systems to avoid cell injury. For example, 17*β*-estradiol upregulates the Nrf2 pathway *via* activation of the estrogen receptor to suppress light-induced rat retinal degeneration (Zhu et al., 2015), and S-(−)equol induces endothelial cell survival by activation of the Nrf2 pathway *via* ER*α* and ER*β* (Zhang et al., 2013). Recent studies have also suggested that the Nrf2 signaling pathway—including Nrf2, heme oxygenase-1 (HO-1), and NAD(P)H:quinone oxidoreductase-1 (NQO-1) expression levels—and SOD activity are decreased, whereas MDA content was increased in injury models using TM4 mouse Sertoli cells that are induced by zearalenone, cadmium, and 1,3-dinitrobenzeneb (Oh et al., 2010; Long et al., 2017; Yang et al., 2018). Collectively, we speculated that ER*α*-mediated signaling *via* Nrf2 was likely to be a potent positive regulator in the maintenance of Sertoli cell survival and function. Thus, therapeutic approaches targeting the ER*α*/Nrf2 signaling pathway could represent a novel strategy to counteract Sertoli cell injury with aging.

*Epimedium*, a traditional Chinese herb, has been widely used in China for thousands of years as a natural drug for male reproductive dysfunction and aging (Chinese Pharmacopoeia Commission, 2015), and its principal bioactive ingredient is icariin (Wu et al., 2012; Chen et al., 2014). Icariin has been reported to promote proliferation of primary rat Sertoli cells (Nan et al., 2014) and attenuate microcystin-LR-induced Sertoli cell injury *in vitro* (Zhou et al., 2019). Importantly, icariin improved Sertoli cell function and increased epididymal sperm count in male rats (Chen et al., 2014). These results suggest that icariin plays important roles in protection from Sertoli cell injury. However, the protective effects of icariin on an age-related decline in testicular function and its underlying mechanisms of action remain unclear. In particular, the possible protective effects of icariin on Sertoli cell injury *via* the ER*α*/Nrf2 signaling pathway are poorly understood.

In the present study, we therefore evaluated the protective effect of icariin on the decline in testicular function using a naturally aging rat model. We then elucidated the protective effects of icariin on Sertoli cell injury as mediated by the ER*α*/Nrf2 signaling pathway *in vivo* and *in vitro*. We expect that our present findings will lead to a new preventive strategy to be used against age-related decline in testicular function.

## Materials and Methods

### Icariin and DMSO

Icariin was purchased from Chengdu PureChem-Standard Co., Ltd (No. 160920; Chengdu, Sichuan, China), and the purity was 98.61%. For the *in vitro* experiment, icariin was dissolved in dimethyl sulfoxide (DMSO) as a stock solution (50 µM) and stored at 4°C; serial dilutions were made in DMSO.

### Animals and Treatments

Male Sprague-Dawley (SD) rats at 2 and 16 months of age were purchased from Beijing Vital River Laboratory Animal Technology Co., Ltd. (Beijing, China) and fed by the Animal Center of China Three Gorges University, Yichang, China. All animal protocols were approved by the China Three Gorges University Council on Animal Care Committee and maintained in accordance with the guidelines and present regulations of our Institutional Animal Ethics Committee.

Rats were randomly divided into four groups: an adult control group (2 months of age), an aging model group (16 months of age), and low and high doses of icariin-treated groups (16 months of age), with 10 rats in each group. The adult control and aging model groups only received a normal diet, while icariin-treated groups were given a diet containing icariin at two different doses (2 and 6 mg/kg) for 4 months. After treatment, all rats were euthanized by exsanguination under urethane anesthesia. We then removed and weighed the testis and epididymis and calculated the gonadal index using the following formula: gonad index (mg/g) = testicular or epididymal weight (mg)/body weight (g). The right testis from each rat was stored at −80°C until further analysis, while the left testis was fixed in 4% paraformaldehyde for histologic evaluation and immunofluorescence analysis. The right epididymis was taken for analysis of sperm count and viability, per our previous publication (Zhao et al., 2019).

### Evaluation of Testosterone and Estradiol Concentrations

The levels of testosterone and estradiol in testes were determined using ELISA kits according to the manufacturer’s instructions (R&D Systems, MN, USA).

### Histologic Evaluation

Paraffin-embedded testicular tissues were sectioned to a thickness of 5 μm, stained with hematoxylin and eosin (H&E) using standard protocols, and observed under light microscopy. We measured seminiferous tubule diameter and seminiferous tubule height using ImageJ software (NIH, Bethesda, MD, USA) for more than 200 seminiferous tubules per animal, with 5–8 rats in each group.

### Transmission Electron Microscopy

Fresh testicular tissues were immersed in 2.5% glutaraldehyde for 2 h and post-fixed in 1% osmium tetroxide for 1 h. Then, the tissues were rehydrated, embedded, sectioned, and double-stained with uranyl acetate and citrate. We recorded the images of Sertoli cell ultrastructure under a Hitachi H-7500 transmission electron microscope (Hitachi Ltd., Tokyo, Japan).

### Analysis of SOD Activity and MDA Content

Frozen right testicular tissues were rinsed and homogenized using ice-cold isotonic saline, and the homogenates (10% w/v) were centrifuged at 3,000 × g at 4°C for 10 min. We then determined the concentrations of protein in the supernatants with a BCA protein assay kit. SOD activity and MDA content in testicular tissues were assayed according to the manufacturer’s instructions.

### Cell Culture and Transfection

We purchased the mouse Sertoli cell line TM4 from ATCC (Manassas, VA, USA). Cells were cultured in F-12/DMEM (Gibco) containing 10% fetal bovine serum (FBS, Gibco) at 37°C in a humidified incubator with 5% CO_2_ in compressed air.

Three predesigned ER*α*-specific small interfering RNAs (siRNAs) and a scramble control siRNA (GenBank accession number, NM_007956.5) were designed by GenePharma Co., Ltd., Shanghai, China, and specificity for ER*α* disruption was determined by transfecting the three siRNAs into TM4 cells according to the manufacturer’s protocol. After screening to validated potential siRNAs, the ER*α* siRNA target sequence (forward primer, 5′-CCGGCACAUGAGUAACAAATT-3′; reverse primer, 5′-UUUGUUACUCAUGUGCCGGTT-3′) was selected for further study. Briefly, TM4 cells at a concentration of 5 × 10^5^ cells/well in 6-well plates were transfected with ER*α* siRNA at different concentrations (50, 100, and 150 nM) or nontargeting scramble control siRNA (forward primer, 5′-UUCUCCGAACGUGUCACGUTT-3′; reverse primer, 5′-ACGUGACACGUUCGGAGAATT-3′) using EntransterTM-R4000 for 60 h; transfection efficiency was monitored by determining the protein expression levels of ER*α* using western immunoblotting analysis.

### Effects of Icariin on TM4 Cells Injury Induced by D-Galactose (D-gal) *In Vitro*

The senescence model of attenuated secretory function of TM4 cells was induced by D-gal (No. G5388; Sigma-Aldrich, USA) *in vitro* as we have described previously (Chen et al., 2018). Preliminary results showed that icariin at concentrations of 0.01–1 μM were nontoxic to D-gal (100 mM)-stimulated TM4 cells using the methylthiazoletetrazolium (MTT; No. 0793; Solarbio, USA) assay for determination of cellular viability. In addition, icariin at 0.5 and 1 μM promoted cellular viability. Therefore, in the present study, we applied icariin at 0.5 and 1 μM concentrations.

TM4 cells numbering 5 × 10^5^/well in 6-well plates were pretreated with icariin (0.5 and 1 μM) or 0.1% DMSO for 20 h and then cultured with D-gal (100 mM) for 60 h. The cells were then harvested for protein expression and immunofluorescence analysis.

To further evaluate whether ER*α* signaling was involved in the protective action of icariin in TM4 cell injury, the confluent TM4 cells transfected with 100 nM ER*α* siRNA were treated with icariin (1 μM) or 0.1% DMSO for 20 h and then incubated with D-gal (100 mM) for 60 h; the relative protein expression levels of GDNF, PLZF, BMP4, SCF, ER*α*, Nrf2, HO-1, and NQO-1 in TM4 cells were determined.

### Analysis of Senescence-Associated *β*-Galactosidase (SA-*β*-gal) Staining

TM4 cells as described above were washed twice with phosphate-buffered saline (PBS), and then the activity of SA-*β*-gal was measured using the senescence-associated *β*-galactosidase staining kit (Beyotime Institute of Biotechnology, Shanghai, China). We then determined the percentage of SA-*β*-gal-positive cells by counting a minimum of 500 cells.

### Measurement of Intracellular ROS

The production of intracellular ROS was detected using the DCFH-DA fluorescence assay (No. 4091-99-0; Sigma-Aldrich, USA). Briefly, TM4 cells as described above were harvested, incubated with 500 μl of ROS detection solution at 37°C for 15 min in the dark and then analyzed by flow cytometry (BD FACSVerse).

### Western Immunoblotting Analysis

Total proteins were extracted from testicular tissues and TM4 cells as described above, and the relative protein expression levels of GDNF, PLZF, BMP4, SCF, ER*α*, Nrf2, HO-1, NQO-1, and *β*-actin were measured using western blotting. Antibodies used in this experiment are presented in **Supplementary Table 1**.

### Immunofluorescence Analysis

Each slide with several samples of testicular paraffin sections was deparaffinized, rehydrated, and incubated in boiling citric acid buffer (pH 6.0) for 10 min for antigen retrieval. After cooling, we incubated sections in hydrogen peroxide buffer (10%) for 15 min to block endogenous peroxidase activity and then blocked them with 5% bovine serum albumin (BSA) at room temperature for 1 h. For analysis, the aforementioned numbers of Sertoli cells were incubated with primary antibody against SOX9 (Sertoli cell marker) overnight at 4°C. After three washes with PBS, we incubated sections with Alexa Fluor 594 donkey antirabbit secondary antibody for 1 h at room temperature. After three additional washes with PBS, cell nuclei were counter-stained with 4′,6-diamidino-2-phenylindole (DAPI, Invitrogen Corporation, USA) in PBS for 10 min. We counted Sertoli cell numbers using Image J software for more than 80 tubules per animal, with 5–8 animals per experimental group. For double immunofluorescence analysis, cells were first incubated with primary antibody against SOX9 overnight at 4°C and then incubated with Alexa Fluor 594 donkey antirabbit lgG (H + L) for 1 h at room temperature. Next, we incubated sections with ER*α*, ER*β*, or Nrf2 antibodies overnight at 4°C, incubated them with Alexa Fluor 488 donkey antirabbit secondary antibodies for 1 h at room temperature, and then counter-stained them with DAPI. The fluorescence images were observed using a Nikon A1R+ confocal microscope (Nikon Corporation, Chiyoda, Japan).

TM4 cells were fixed in 4% paraformaldehyde for 30 min and treated with 0.1% Triton X-100 (Sigma, USA) for 15 min at room temperature. We immersed cells in 1% BSA in PBS at room temperature for 1 h and incubated them with Nrf2 antibody overnight at 4°C. Following three washes with PBS, the slides were exposed to Alexa Fluor 488 donkey antirabbit secondary antibodies for 1 h at room temperature and then washed with PBS. Cell nuclei were stained with DAPI for 5 min. We ultimately examined Nrf2 expression and location under a Nikon A1R+ confocal microscope.

### Statistical Analysis

The results are expressed as means ± SEM. A *P* value of <0.05 was considered statistically significant. The differences between two groups were analyzed using the unpaired Student’s t test, and the differences between multiple groups were analyzed using one-way ANOVA, followed by Dunnett’s multiple-range test for multiple comparisons using GraphPad Prism 7 Software.

## Results

### Icariin Ameliorates the Decline in Testicular Function in Aging Rats

**Figure 1:**
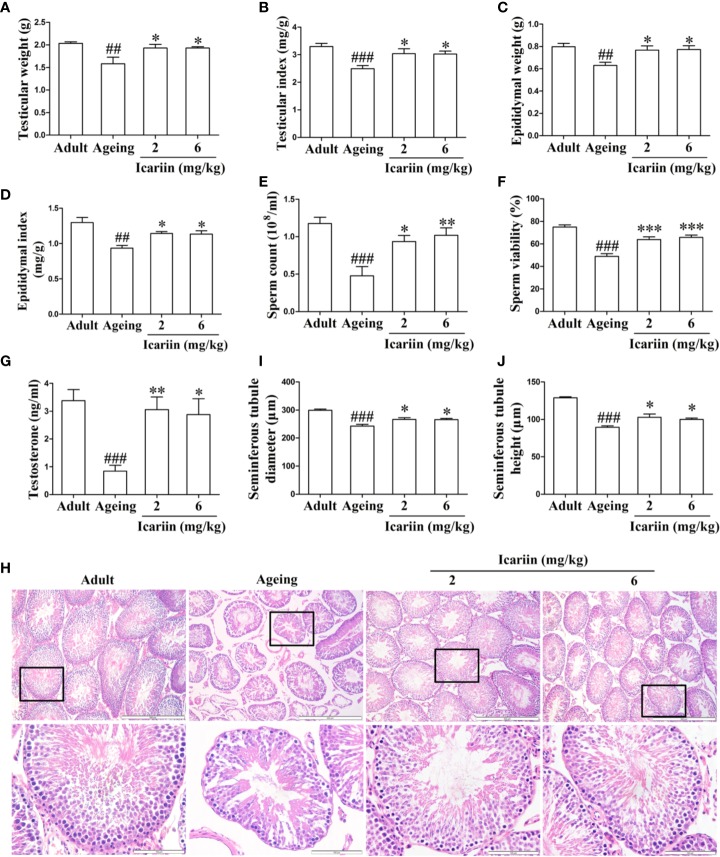
Icariin ameliorates the decline in testicular function in aging rats. **(A)** Testicular weight. **(B)** Testicular index. **(C)** Epididymal weight. **(D)** Epididymal index. **(E)** Sperm count. **(F)** Sperm viability. **(G)** Testicular testosterone concentrations. **(H)** Testicular histology. Representative images of haematoxylin and eosin staining. Upper panels indicate the alterations of several seminiferous tubules (original magnification, 100×; scale bar = 500 μm). Lower panels correspond to magnified boxed areas (original magnification, 400×; scale bar = 100 μm). **(I)** Seminiferous tubule diameter. **(J)** Seminiferous tubule height. Seminiferous tubule diameters and seminiferous tubule heights were measured using Image J software for more than 200 tubules per animal, with 5–8 rats in each group. Data are presented as means ± SEM. ^#^*P*< 0.05, ^##^*P* < 0.01, ^###^*P* < 0.001 *versus* adult control group; ^*^P < 0.05, ^**^P < 0.01, ^***^P < 0.001 *versus* aging model group.

To investigate whether icariin ameliorates the decline in testicular function with increasing age, we first measured the weights and other indices of the testis and epididymis for each group. As shown in **Figures 1A–D**, testicular and epididymal weights and their indices in the aging model group were significantly decreased compared to the adult control group. However, icariin significantly increased testicular and epididymal weights and their indices in aging rats. Furthermore, icariin markedly increased sperm count and sperm viability (**Figures 1E, F**). We then assayed the levels of testicular testosterone and found that they were significantly decreased in the aging model group compared to the adult control group (**Figure 1G**). Conversely, icariin significantly increased testosterone levels.

Finally, to further clarify the preventive effect of icariin on the decline in testicular function with aging, we assessed histologic alterations in the testis. Tissue sections from adult rats showed that germ cells at different stages in seminiferous tubules were arranged in typical fashion and that spermatogonia and Sertoli cells were at the basement membrane (**Figure 1H**). However, the numbers of germ cells were reduced and most of the seminiferous tubules were loosely packed in aging rats. Further analysis revealed that seminiferous tubule diameters and heights were significantly decreased in the aging model group, indicative of fewer germ cells in the tubules (**Figures 1I, J**). In contrast, administration of icariin delayed the degeneration of spermatogenesis with aging and significantly increased tubular diameters and epithelial heights. These results suggested that icariin was effective in delaying functional decline in the testis of aged rats.

### Icariin Alleviates Sertoli Cell Injury *In Vivo* and *In Vitro*

**Figure 2:**
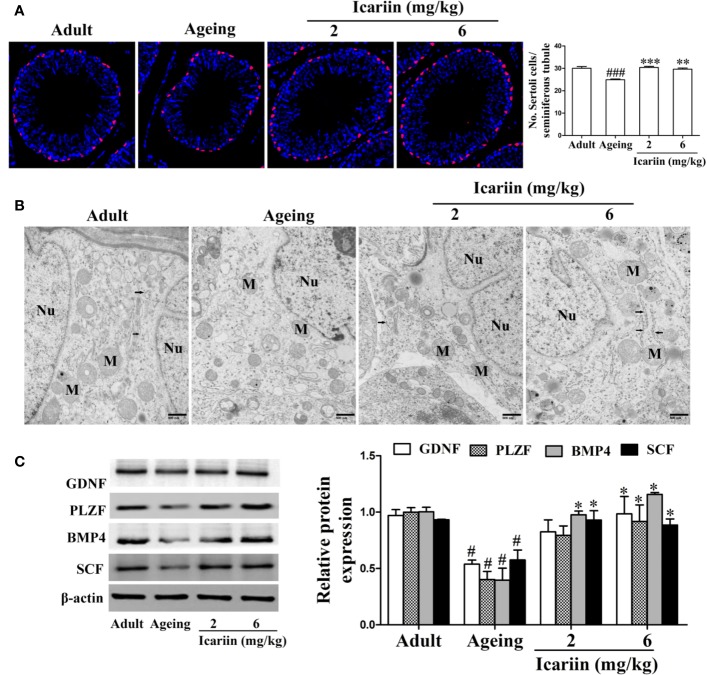
Icariin protects against testicular Sertoli cell injury in aging rats. **(A)** The numbers of Sertoli cells in testicular tissue. Anti-SOX9 rabbit-polyclonal antibody (red) was used to detect Sertoli cell numbers, and DAPI (blue) was used for nuclear staining (original magnification, 400×). Sertoli cell numbers were measured using Image J software for more than 80 tubules per animal, with 5–8 rats in each group. **(B)** The ultrastructure of Sertoli cell in testicular tissues was examined using a Hitachi H-7500 transmission electron microscopy (original magnification, 20,000×; scale bar = 800 nm). These electronmicrographs are representative of three independent experiments. Nu, nuclei; M, mitochondria. Black arrowheads indicate endoplasmic reticulum. **(C)** The relative protein expression levels of GDNF, PLZF, BMP4, and SCF in testicular tissues were detected using western immunoblotting analysis. All values are expressed as means ± SEM of three independent experiments. ^#^*P* < 0.05, ^###^*P* < 0.001 *versus* adult control group; ^*^P < 0.05, ^**^P < 0.01, ^***^P < 0.001 *versus* aging model group.

Sertoli cells play an important role in the regulation of spermatogenesis (Johnson et al., 2008). However, Sertoli cells are negatively impacted by aging, including manifesting abnormal morphologic structure, number, and function (Johnson et al., 1984; Paniagua et al., 1985; Tenover et al., 1988; Anderson, 2000; Jiang et al., 2014). To determine whether icariin protects against Sertoli cell injury with aging, we first used SOX9 as a Sertoli cell marker to measure Sertoli cell numbers in the testis from aged rats. As shown in **Figure 2A**, we found that the numbers of Sertoli cells per seminiferous tubule were significantly reduced in the aging group when compared with the adult controls; in contrast, the numbers of Sertoli cells in the icariin-treated group were significantly increased. We then investigated the effects of icariin on the ultrastructure of Sertoli cells in aging rats, which shows in **Figure 2B** that Sertoli cells in aging rats contained abundant cytoplasmic vacuoles, expanded endoplasmic reticula, and numerous mitochondria displaying tubular cristae. However, icariin, to some extent, improved the ultrastructure of Sertoli cells. Finally, we determined the protective effects of icariin on Sertoli cell function using western blotting analysis and found that icariin significantly increased the relative protein expression levels of GDNF, PLZF, BMP4, and SCF (**Figure 2C**).

**Figure 3:**
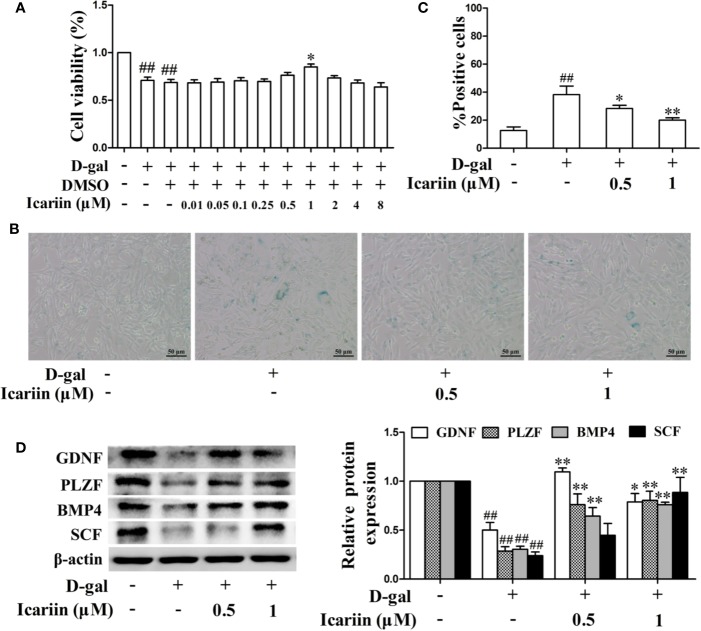
Icariin alleviates TM4 cells injury induced by D-galactose (D-gal). **(A)** Cellular viability was detected using MTT assay. TM4 cells at a concentration of 3 × 10^4^ cells/well in 96-well plates were pretreated with icariin (0.01–8 μM) or DMSO (0.1%) for 20 h and then incubated with 100 mM D-gal for 60 h. **(B–D)** TM4 cells at 5 × 10^5^/well in 6-well plates were pretreated with icariin (0.5–1 μM) for 20 h and then incubated with 100 mM of D-gal for 60 h. **(B)** Representative images of SA-*β*-gal staining of cells (scale bar = 50 μm). **(C)** The percentages of SA-*β*-gal-positive cells from a total of 500 cells. **(D)** The relative protein expression levels of GDNF, PLZF, BMP4, and SCF were measured using western blotting analysis. All values are expressed as means ± SEM of three independent experiments. ^##^*P* < 0.01 *versus* control; ^*^P < 0.05, ^**^P < 0.01 *versus* D-gal-stimulated group.

The number and function of cells in the mouse Sertoli cell line TM4 were decreased by D-gal (**Supplementary Figures S1** and **S2**) (Chen et al., 2018), suggesting that these cells might be a model for Sertoli-cell injury associated with aging. Therefore, we used the models to evaluate whether icariin attenuates Sertoli cell injury and found that icariin increased cellular viability in a concentration-dependent manner at 0.01–1 μM, and then caused cellular toxicity (**Figure 3A**), suggesting icariin displayed hormetic dose response characterized by a low-dose stimulation and a high-dose inhibition. Thus, we selected 0.5 and 1 μM icariin in the next experiments. As shown in **Figures 3B, C**, we found that icariin significantly inhibited cellular senescence of TM4 cells stimulated by D-gal. As expected, icariin also significantly increased the function of D-gal-stimulated TM4 cells as evidenced by western blotting analysis (**Figure 3D**). These data indicated that icariin protected Sertoli cells from injury due to aging.

### ER*α* Signaling Is Involved in the Protective Action of Icariin in Sertoli Cell Injury

**Figure 4:**
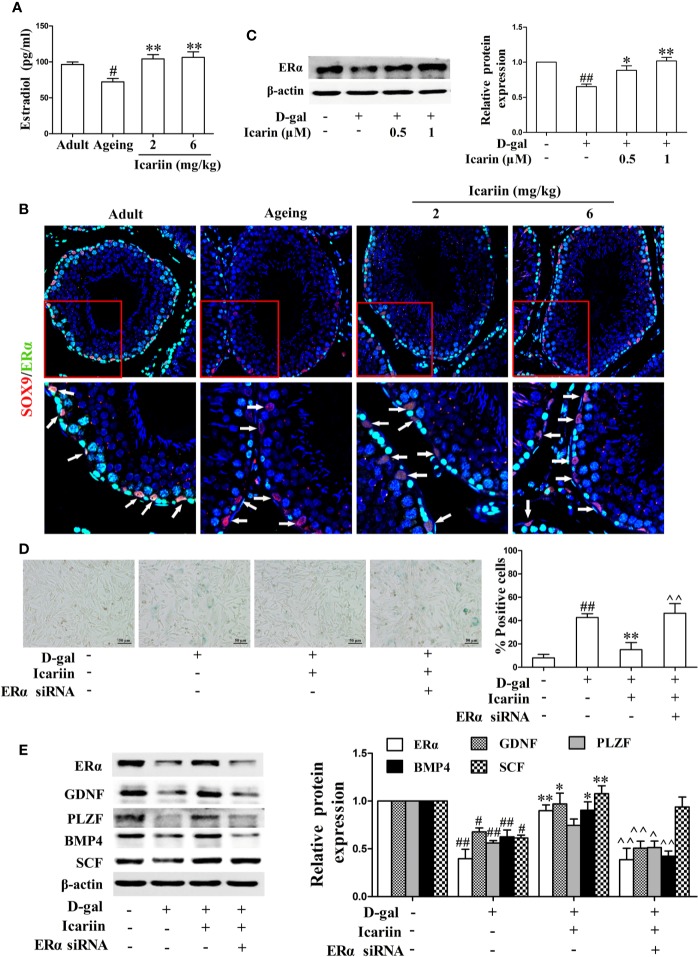
Icariin alleviates Sertoli cell injury *via* ER*α* signaling. **(A)** Testicular estradiol concentrations were measured using ELISA kits. Each value represents the mean ± SEM with 5–6 rats per group. ^#^*P* < 0.05, *versus* adult control group; ^**^P < 0.01 *versus* aging model group. **(B)** Co-localization of Alexa Fluor 488-labeled ER*α* (green) with Alexa Fluro 594-labeled SOX9 (red) in the testis was detected with a Nikon A1R+ confocal microscope. Upper panels indicate one intact seminiferous tubule (original magnification, 400×). Lower panels correspond to magnified boxed areas (original magnification, 800×). White arrows indicate colocalization of ER*α* and SOX9 in Sertoli cells. These photomicrographs are representative of three independent experiments. **(C)** The relative protein expression levels of ER*α* in D-gal-stimulated TM4 cells were measured by western blotting. TM4 cells at 5 × 10^5^/well in 6-well plates were pretreated with or without icariin (0.5–1 μM) or DMSO (0.1%) for 20 h, and then incubated with 100 mM D-gal for 60 h. All values are expressed as means ± SEM of three independent experiments. **(D, E)** TM4 cells at 1 × 10^6^/well in 6-well plates transferred with or without ER*α* siRNA were incubated with icariin (1 μM) or DMSO (0.1%) for 20 h, followed by treatment with 100 mM D-gal for 60 h. **(D)** Representative images of SA-*β*-gal staining of cells (left; scale bar = 50 μm), and the percentages of SA-*β*-gal-positive cells from a total of 500 cells (right). **(E)** The relative protein expression levels of ER*α*, GDNF, PLZF, BMP4, and SCF in TM4 cells were measured by western blotting analysis. All values are expressed as means ± SEM of three independent experiments. ^#^*P* < 0.05, ^##^*P* < 0.01 *versus* control; ^*^P < 0.05, ^**^P < 0.01 versus D-gal-stimulated group; ^^^*P* < 0.05, ^^^^*P* < 0.01 *versus* icariin-treated group.

Estrogen and its receptor ER*α* have been reported to promote Sertoli cell proliferation and facilitate Sertoli cell function during the process of spermatogenesis (Sneddon et al., 2005; Sinkevicius et al., 2009; Royer et al., 2012). Therefore, we investigated whether icariin affected estrogen levels and ER*α* expression in the testis. As shown in **Figures 4A, B**, the levels of estradiol in the aging testis were significantly diminished, and the expression of ER*α* in Sertoli cells appeared to be attenuated in the aging group compared to the adult controls. Conversely, icariin significantly increased estradiol levels and enhanced ER*α* expression in Sertoli cells. In addition, icariin enhanced ER*α* expression in spermatogonia and primary spermatocytes, suggesting that estrogen and its receptor ER*α* are important for the maintenance of normal spermatogenesis and overall homeostasis.

To further test the effects of icariin on ER*α* signaling in Sertoli cells, we also examined the alterations in ER*α* expression levels in D-gal-stimulated TM4 cells. As shown in **Figure 4C**, icariin significantly increased the relative protein expression levels of ER*α*. To further test whether ERα was involved in the protective effects of icariin on Sertoli cell injury, we transfected TM4 cells with siRNA against ER*α* and found that ER*α* silencing abrogated the icariin-mediated positive effect (**Figures 4D, E**). These results indicated that icariin attenuated Sertoli cell injury, at least partially *via* upregulation of ER*α* signaling.

### Nrf2 Signaling Pathway Is Also Involved in the Protective Action of Icariin on Sertoli Cell Injury

**Figure 5:**
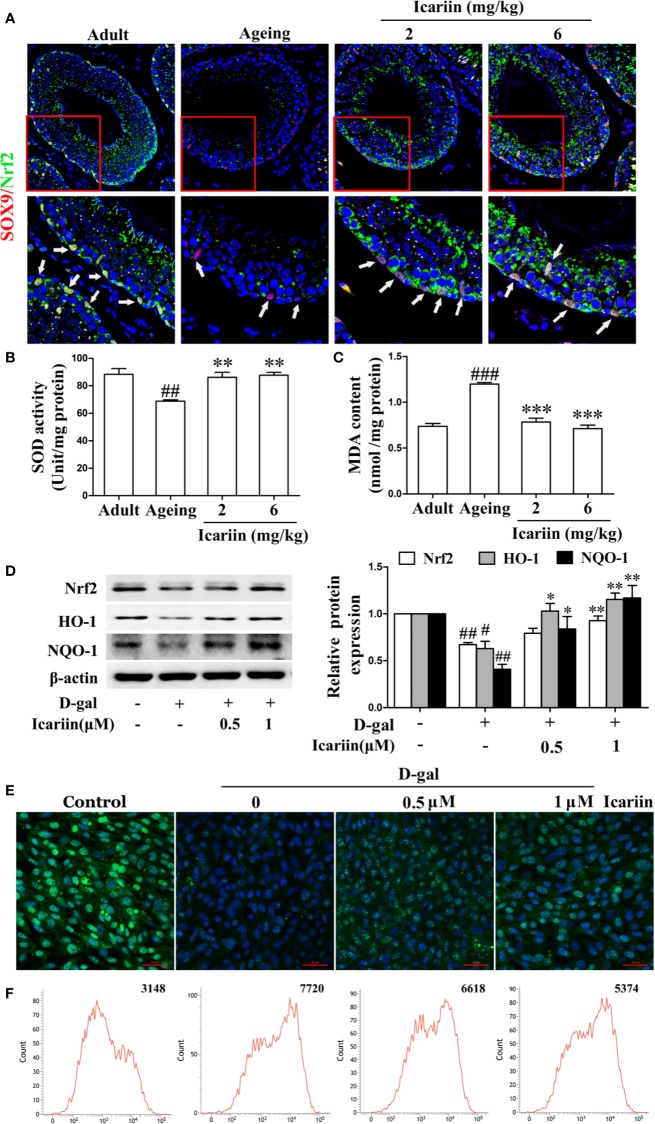
Icariin activates Nrf2 signaling in Sertoli cells. **(A)** Colocalization of Alexa Fluor 488-labeled Nrf2 (green) with Alexa Fluro 594-labeled SOX9 (red) in testis was examined with a Nikon A1R+ confocal microscope. Upper panels indicate one intact seminiferous tubule (original magnification, 400×). Lower panels correspond to magnified boxed areas (original magnification, 800×). White arrows indicate co-localization of Nrf2 and SOX9. These photomicrographs are representative of three independent experiments. **(B)** Activity of SOD. **(C)** Content of MDA. Each value represents the means ± SEM, with 5–6 rats in each group. ^##^*P* < 0.01, ^###^P < 0.001 *versus* adult control group; ^**^P < 0.01, ***P < 0.001 *versus* aging model group. **(D–F)** TM4 cells were pretreated with icariin (0.5–1 μM) or DMSO (0.1%) for 12 h, and then incubated with 100 mM D-gal for 60 h. **(D)** The relative protein expression levels of Nrf2, HO-1, and NQO-1 in TM4 cells were measured by western blotting analysis. **(E)** Representative images of Nrf2 using immunofluorescence (scale = 50 μm). **(F)** Intracellular ROS levels were measured using flow cytometry. All values are expressed as means ± SEM of three independent experiments. ^#^*P* < 0.05, ^##^*P* < 0.01 *versus* control; ^*^P < 0.05, ^**^P < 0.01 *versus* D-gal-stimulated group.

It has been reported that inhibition of the Nrf2-signaling pathway causes Sertoli cell injury (Oh et al., 2010; Long et al., 2017; Yang et al., 2018). In testicular Sertoli cells *in vivo* we found that icariin indeed upregulated Nrf2 expression in Sertoli cells of aging rats (**Figure 5A**). We also found that icariin increased the expression of Nrf2 in spermatogenic cells, augmented SOD activity, and decreased MDA content in the testis (**Figures 5A–C**). Similarly, icariin significantly promoted Nrf2 nuclear translocation and increased protein expression levels of Nrf2 and downstream signaling molecules such as HO-1 and NQO-1 in D-gal-stimulated TM4 cells (**Figures 5D, E**). Commensurately, the production of ROS was inhibited by icariin in D-gal-stimulated TM4 cells (**Figure 5F**). Interestingly, TM4 cells underwent cell death when Nrf2 was silenced after treatment with D-gal. Thus, we tested the functional changes in TM4 cells transfected with Nrf2 siRNA without D-gal and icariin treatment and found that Nrf2 silencing significantly inhibited Sertoli cell function (**Supplementary Figure S3**). These results indirectly indicated that icariin attenuated Sertoli cell injury by upregulating Nrf2 signaling.

### Icariin Alleviates Sertoli Cell Injury *via* the ER*α*/Nrf2-Signaling Pathway

**Figure 6:**
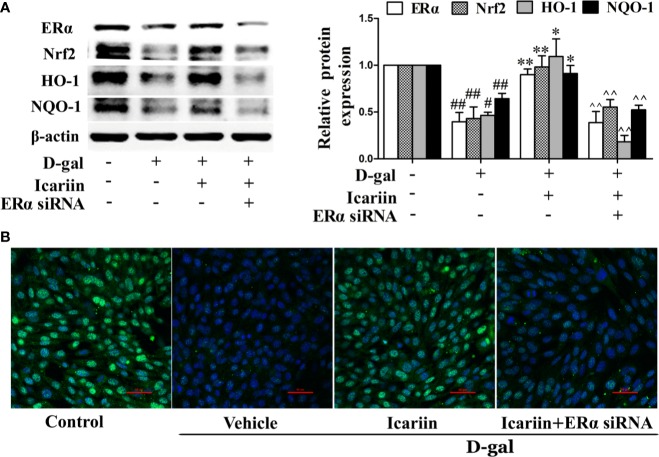
Icariin protects against TM4 cells injury *via* the ER*α*/Nrf2 signaling pathway. TM4 cells at 1 × 10^6^/well in 6-well plates transferred with or without ER*α* siRNA were incubated with icariin (1 μM) or DMSO (0.1%) for 12 h, followed by treatment with 100 mM D-gal for 60 h. **(A)** The relative protein expression levels of ERα, Nrf2, HO-1, and NQO-1 in TM4 cells as described above were measured by western blotting analysis. All values are expressed as means ± SEM of three independent experiments. ^#^*P* < 0.05, ^##^*P* < 0.01 *versus control*; ^*^P < 0.05, ^**^P < 0.01 *versus* D-gal-stimulated group; ^^P < 0.01 versus icariin-treated group. **(B)** Representative images of Nrf2 using immunofluorescence (scale = 50 μm). These photomicrographs are representative of three independent experiments.

ERα has been demonstrated to protect cells from injury through Nrf2 activation (Ishii and Warabi, 2019). Thus, we further wished to uncover whether icariin attenuated Sertoli cell injury *via* ER*α*-mediated activation of the Nrf2-signaling pathway. We found that ER*α* siRNA significantly decreased Nrf2 expression (**Supplementary Figure S3**) and reversed the increase in Nrf2 signaling activated by icariin in D-gal-stimulated TM4 cells (**Figure 6**). Collectively, these data suggested that icariin alleviated Sertoli cell injury by activating Nrf2 signaling *via* ERα.

## Discussion

We made two novel observations in our study. First, we found that icariin effectively ameliorated age-related degeneration of testicular function through alleviation of Sertoli cell injury, which is the first time that this has been demonstrated. Second, we are the first to demonstrate that icariin protects against Sertoli cell injury by activating the ER*α*/Nrf2-signaling pathway. These results suggest that icariin displays effective preventive effects in age-related testicular dysfunction by alleviating Sertoli cell injury and is dependent upon the ER*α*/Nrf2 pathway.

Previous studies have indicated that testicular spermatogenic function declines with aging—including the loss of germ cells’ number, the decline of Sertoli cells and Leydig cells’ number and function leading to the disruption of SSCs’ niche, the recession of sex hormones levels (Haji et al., 1994; Petersen et al., 2015; Li et al., 2017; Wang et al., 2017). Clinical studies have shown that testosterone concentration, sperm count and quality are declined, and the incidence of infertility is increased in aged men (Gunes et al., 2016; Almeida et al., 2017). In our study, we found icariin effectively ameliorated the age-related decline in testicular function by increasing testicular and epididymal weights and indices, sperm count and sperm viability, testicular testosterone concentrations, and seminiferous tubule diameters and heights. The promotion of icariin on testosterone secretion in our study is consistent with previous reports (Zhang and Yang, 2006; Chen et al., 2014). Testosterone is mainly synthesized and secreted by Leydig cells in testes, and one of mechanism of icariin action on the reproductive function of male rats is the testosterone mimetic properties (Zhang and Yang, 2006). Investigators have found that icariin promotes testosterone secretion not only by increasing cAMP levels *via* upregulation of luteinizing hormone receptor expression in Leydig cell (Xiong and Zhou, 1994; Chen et al., 2014), but also by upregulating the mRNA expression of key enzymes involved in the conversion of cholesterol to testosterone, such as StAR, Cyp17a1, and PBR (Chen et al., 2014). Consequently, intratesticular testosterone can bind with androgen receptor in Sertoli cells and peritubular myoid cells to control secretion of proteins from Sertoli cells—eventually support development of pachytene spermatocytes and germ cells through stage VII (Weinbauer et al., 2004). Thus, we speculated icariin can indirectly improve Sertoli cell function and testicular degeneration during aging by promoting testosterone secretion.

Sertoli cells are believed to be “nurse cells” or “mother cells” and play pivotal roles in testis formation and spermatogenesis (Griswold, 1998). They facilitate the progression of germ cells to spermatozoa *via* direct contact by establishing and maintaining the SSC niche—which ensures stem cell renewal and spermatogonial differentiation toward mature germ cells. In the process of spermatogenesis, a number of soluble factors are specifically produced by Sertoli cells—including GDNF, PLZF, BMP4, and SCF—that are essential for self-renewal and differentiation of SSCs. Increasing evidence suggests that mice with mutant GDNF or deletion of GDNF and its receptors Ret and Gfra1 in SSCs result in stem cell loss, severe fertility defects, and spermatogenic disruption (Meng et al., 2000; Naughton et al., 2006). However, GDNF production was decreased with aging accompanied by the loss of SSC numbers (Ryu et al., 2006), which demonstrates that decline in Sertoli cell function is an important factor in age-related dysfunction of the testis. Furthermore, reduced Sertoli cell number and abnormal morphologic structure were observed with aging (Johnson et al., 1984; Filipiak et al., 2013). However, there is little information on the protective effects of dietary flavonoids on Sertoli cell injury with advancing age. Our results showed that icariin, a flavonoid from *Epimedium*, attenuated Sertoli cell injury *in vivo* and *in vitro*, thereby attenuating age-related degeneration of testicular function. Notably, icariin’s protective effects in Sertoli cell injury and testicular degeneration were not significantly different between the two groups (shown in **Figures 1** and **2**), suggesting that icariin displays hormetic dose response features *in vivo*. Given hormetic dose response is a biphasic dose response phenomenon characterized by a low-dose stimulation and a high-dose inhibition (Calabrese et al., 2010; Calabrese et al., 2018; Scuto et al., 2019), the dose range of icariin on protection against Sertoli cell injury and testicular dysfunction with aging should be further explored to obtain maximum health outcomes in future.

The estrogen/ER*α*-signaling pathway is known to be one of the major modulators in maintaining optimal levels of spermatogenesis in adults (Lucas et al., 2011; Clarke and Pearl, 2014). It was reported that bioavailable estradiol was reduced with aging in humans (Carreau et al., 2004) and that human testis express ERα (Filipiak et al., 2013). Furthermore, estradiol levels and ER*α* expression levels were reported to decline in the testis of aging rats (Hamden et al., 2008; Clarke and Pearl, 2014). Increasing evidence indicates that ER*α* knockout induces testicular atrophy and infertility in mice (Hess et al., 2011), and that ER*α* inhibition also decreases sperm motility and fertility in mice and rats (Oliveira et al., 2001; Cho et al., 2003; Johnson et al., 2008). In contrast, exogenous estradiol treatment attenuated the age-related loss of sperm production in rats by increasing ER*α* expression, while ER*β* expression was not affected during aging or treatment with exogenous estradiol (Clarke and Pearl, 2014). Given that icariin promoted MC3T3-E1 osteoblastic cell and UMR 106 cell proliferation and function *via* activation of estrogen receptors (Mok et al., 2010; Song et al., 2013), we further investigated whether icariin activated the estrogen/ER*α* pathway in injured Sertoli cells. Our results showed that icariin significantly increased ER*α* expression levels, thereby inhibiting injury of testicular Sertoli cells and D-gal-stimulated TM4 cells. Conversely, siRNA ERα partially reversed the icariin (1 µM)-mediated protective effects on Sertoli cell injury *in vitro*. These data indicate that the protection of Sertoli cell injury by icariin is partially dependent upon the estrogen/ER*α* pathway in age-related testicular dysfunction.

Endogenous cellular defense mechanisms are adaptive stress response pathways against oxidative stress to maintain cell function and survival. Among these cellular pathways, upregulation of Nrf2-signaling pathway and the vitagene network, which includes HO-1, contributes to counteract the ROS and excessive oxidative stress (Calabrese et al., 2018). Conversely, Nrf2 pathway inactivation drivers a vicious and self-propagating cycle of redox stress imbalance that causes cell dysfunction even apoptosis. Thus, pharmacotherapeutic approaches that activation of Nrf2 pathway and upregulation of vitagene network are currently considered very promising for the treatment of various age-related diseases (Trovato et al., 2016; Trovato et al., 2018). Recent studies have shown that activation of the Nrf2 pathway protected against zearalenone-induced TM4 cell apoptosis and cadmium-induced testicular Sertoli cell injury in mice (Long et al., 2017; Yang et al., 2018), whereas inhibition of the Nrf2 pathway induced testicular Sertoli cell apoptosis in mice (Wang et al., 2018). In addition, Nrf2 knockout induced ROS accumulation by disrupting redox homeostasis to promote age-related muscle disorders in aged mice (Miller et al., 2012). Thus, we examined the effects of icariin on the Nrf2 pathway and found that it upregulated the expression of Nrf2 and its downstream molecules HO-1 and NQO-1 in testicular Sertoli cells and D-gal-stimulated TM4 cells. Furthermore, icariin increased SOD activity, decreased MDA content in the aging testis, and inhibited ROS production in D-gal-stimulated TM4 cells. Similarly, Nrf2 siRNA significantly decreased TM4 cell function (**Supplementary Figure S3**). These results suggest that Sertoli cells suffer from excessive oxidative stress during testicular aging as evidenced by downregulation of Nrf2 pathway and vitagenes, and upregulation of ROS, but icariin attenuates Sertoli cell injury by inducing upregulation of Nrf2 pathway and vitagenes such as HO-1. Similarly to the effect of icariin on Sertoli cell injury, two doses of icariin have similarly antioxidative effects, indicate the possibility that low dose of icariin may induce adaptive stress responses to against oxidative stress-mediated cell injury, whereas high dose of icariin can be toxic to Sertoli cells.

Investigators recently reported that ER*α* protected against H_2_O_2_-induced neurotoxicity in PC12 cells by activating the Nrf2 pathway and upregulating HO-1 and NQO-1 expression and activity (Meng et al., 2014a). Additionally, gypenoside XVII (GP-17), a novel phytoestrogen isolated from *Gynostemma pentaphyllum* or *Panax notoginseng*, conferred protection against A*β*25-35-induced neurotoxicity in PC12 cells through estrogen receptor-dependent activation of Nrf2/ARE/HO-1 pathways (Meng et al., 2014b). The phytoestrogen ginsenoside Rb1 inhibited 6-hydroxydopamine-induced oxidative injury in human dopaminergic cells *via* an estrogen receptor-dependent Nrf2 pathway (Hwang and Jeong, 2010), and a new study indicated that estrogen *via* its receptors induced rapid activation of Nrf2, playing a key role in promoting the proliferation and function of cells in different systems (Ishii and Warabi, 2019). In the present study, we found that ER*α* siRNA reversed icariin-induced activation of the Nrf2-signaling pathway. Thus, we concluded that the ER*α*/Nrf2-signaling pathway contributes to the attenuation of Sertoli cell injury by icariin.

In conclusion, icariin protects against Sertoli cell injury due to age-related testicular dysfunction by upregulating the ER*α*/Nrf2-signaling pathway and vitagene system. These results support the concept of amelioration by icariin of age-related testicular dysfunction. In addition, our findings suggest novel mechanisms by which icariin acts. Our study thus provides, at least in part, a molecular basis for the further application of icariin in the prevention of testicular dysfunction in men with increasing age.

## Data Availability Statement

All datasets generated for this study are included in the article/**Supplementary Material**.

## Ethics Statement

The animal study was reviewed and approved by China Three Gorges University Council on Animal Care Committee.

## Author Contributions

HZ, CZ, and YD: conceived and designed the research. HZ: conducted the experiments and wrote the manuscript. HZ, XY, and QC: worked the experiment. SY, QM, YH, and JW: carried out other experiments and analyzed the data. CL and YD: helped revise the article. All authors revised the manuscript and approved the final version.

## Funding

This work was supported by the National Natural Science Foundation of China (Grant Numbers 81873077, 81774316, 81503334 and 81573931).

## Conflict of Interest

The authors declare that the research was conducted in the absence of any commercial or financial relationships that could be construed as a potential conflict of interest.

## Supplementary Material

Supplementary Figure S1 D-galactose (D-gal) treatment is sufficient to induce TM4 cell senescence. **(A)** Cellular viability was detected using MTT assay. TM4 cells at a concentration of 3 × 10^4^ cells /well in 96-well plates were treated with D-gal at various concentrations (0–250 mM) for 60 h. **(B–D)** TM4 cells at 5 × 10^5^/well in 6-well plates were treated with 100 or 150 mM of D-gal for 60 h. **(B)** Representative images from SA-β-gal staining of cells (left; scale bar = 50 μm) and the percentages of SA-β-gal-positive cells from a total of 500 cells (right). **(C)** The cell cycle was detected by flow cytometry. **(D)** The relative mRNA expression levels of P21 and P16 were measured with RT-PCR. Data are presented as means ± SEM of three independent experiments. **P* < 0.05, ***P* < 0.01 versus control.

Supplementary Figure S2 D-galactose (D-gal) treatment is effective in inducing a decline in TM4 cell function and Nrf2 signaling. TM4 cells at 5 × 10^5^/well in 6-well plates were treated with 100 or 150 mM of D-gal for 60 h. **(A)** The relative mRNA expression levels of GDNF, PLZF, BMP4, and SCF were measured with RT-PCR. **(B)** The relative protein expression levels of GDNF, PLZF, BMP4, and SCF were measured using western immunoblotting analysis. **(D)** The relative protein expression levels of Nrf2, HO-1 and NQO-1 were measured using western blotting analysis. Data are presented as means ± SEM of three independent experiments. **P* < 0.05, ***P* < 0.01 versus control.

Supplementary Figure S3 Nrf2 is involved in the maintenance of Sertoli cell function. TM4 cells at 1 × 10^5^/well in 6-well plates were treated with D-gal (100 mM) for 60 h or transferred with ERα siRNA for 60 h or Nrf2 siRNA for 72 h. **(A)** The relative protein expression levels of ERα and Nrf2 in TM4 cells were measured by western blotting analysis. **(B)** The relative protein expression levels of ERα, GDNF, PLZF, BMP4, and SCF in TM4 cells were measured using western blotting analysis. ^#^*P* < 0.05, ^##^*P* < 0.01, ^###^*P* < 0.01 versus negative control; ^^^*P* < 0.05, ^^^^*P* < 0.01 versus ERα siRNA group.

Suplementary Table S1 Antibodies used in this study.

## References

[B1] Almeida S. Rato L. Sousa M. Alves M. G. Oliveira P. F. (2017). Fertility and sperm quality in the aging male. Curr. Pharm. Des. 23 (30), 4429–4437. 10.2174/1381612823666170503150313 28472913

[B2] Anderson R. A. (2000). Sertoli cell function in the ageing male. Clin. Endocrinol. (Oxf) 53 (2), 139–140. 10.1046/j.1365-2265.2000.01070.x 10931092

[B3] Aydın A. F. Çoban J. Doğan-Ekici I. Doğru-Abbasoğlu S. Uysal M. Koçak-Toker N. (2015). Carnosine and vitamin E-a promising pair in the combat against testicular oxidative stress in aged rats. Andrologia 47 (10), 1131–1138. 10.1111/and.12392 25557643

[B4] Banerjee A. Anjum S. Verma R. Krishna A. (2012). Alteration in expression of estrogen receptor isoforms alpha and beta, and aromatase in the testis and its relation with changes in nitric oxide during aging in mice. Steroids 77 (6), 609–620. 10.1016/j.steroids.2012.02.004 22366072

[B5] Calabrese V. Cornelius C. Dinkova-Kostova A. T. Calabrese E. J. Mattson M. P. (2010). Cellular stress responses, the hormesis paradigm, and vitagenes: novel targets for therapeutic intervention in neurodegenerative disorders. Antioxid. Redox Signal. 13 (11), 1763–1811. 10.1089/ars.2009.3074 20446769 PMC2966482

[B6] Calabrese V. Santoro A. Trovato Salinaro A. Modafferi S. Scuto M. Albouchi F. (2018). Hormetic approaches to the treatment of Parkinson’s disease: Perspectives and possibilities. J. Neurosci. Res. 96 (10), 1641–1662. 10.1002/jnr.24244 30098077

[B7] Carreau S. Bourguiba S. Marie E. (2004). Testicular and blood steroid levels in aged men. Reprod. Biol. 4 (3), 299–304. 15592588

[B8] Chapple S. J. Siow R. C. Mann G. E. (2012). Crosstalk between Nrf2 and the proteasome: therapeutic potential of Nrf2 inducers in vascular disease and aging. Int. J. Biochem. Cell. Biol. 44 (8), 1315–1320. 10.1016/j.biocel.2012.04.021 22575091

[B9] Chen M. Hao J. Yang Q. Li G. (2014). Effects of icariin on reproductive functions in male rats. Molecules 19 (7), 9502–9514. 10.3390/molecules19079502 24995929 PMC6271987

[B10] Chen Q. Zhao H. Ma N. You X. Yang S. Ma Q. (2018). Decline of secretory function of TM4 Sertoli cells stimulated by D-galactose in mice and its mechanism. Chin. J. Cell. Mol. Immunol. 34 (4), 327–333. (in Chinese). 29973323

[B11] Chinese Pharmacopoeia Commission (2015). Pharmacopoeia of People’s Republic of China: One Edition[M]. Beijing: China Med. Sci. Press, 327. (in Chinese).

[B12] Cho H. W. Nie R. Carnes K. Zhou Q. Sharief N. A. Hess R. A. (2003). The antiestrogen ICI 182,780 induces early effects on the adult male mouse reproductive tract and long-term decreased fertility without testicular atrophy. Reprod. Biol. Endocrinol. 1, 57. 10.1186/1477-7827-1-57 12959643 PMC194658

[B13] Clarke M. Pearl C. A. (2014). Alterations in the estrogen environment of the testis contribute to declining sperm production in aging rats. Syst. Biol. Reprod. Med. 60 (2), 89–97. 10.3109/19396368.2014.885995 24499511

[B14] Filipiak E. Suliborska D. Laszczynska M. Walczak-Jedrzejowska R. Oszukowska E. Marchlewska K. (2013). Estrogen receptor alpha localization in the testes of men with normal spermatogenesis. Folia. Histochem. Cytobiol. 50 (3), 340–345. 10.5603/19743 23042285

[B15] França L. R. Hess R. A. Dufour J. M. Hofmann M. C. Griswold M. D. (2016). The Sertoli cell: one hundred fifty years of beauty and plasticity. Andrology 4 (2), 189–212. 10.1111/andr.12165 26846984 PMC5461925

[B16] Griswold M. D. (1998). The central role of Sertoli cells in spermatogenesis. Semin. Cell. Dev. Biol. 9 (4), 411–416. 10.1006/scdb.1998.0203 9813187

[B17] Gunes S. Hekim G. N. Arslan M. A. Asci R. (2016). Effects of aging on the male reproductive system. J. Assist. Reprod. Genet. 33 (4), 441–454. 10.1007/s10815-016-0663-y 26867640 PMC4818633

[B18] Haji M. Tanaka S. Nishi Y. Yanase T. Takayanagi R. Hasegawa Y. (1994). Sertoli cell function declines earlier than leydig cell function in aging Japanese men. Maturitas 18 (2), 143–153. 10.1016/0378-5122(94)90052-3 8177095

[B19] Hamden K. Silandre D. Delalande C. El Feki A. Carreau S. (2008). Age-related decrease in aromatase and estrogen receptor (ERalpha and ERbeta) expression in rat testes: protective effect of low caloric diets. Asian. J. Androl. 10 (2), 177–187. 10.1111/j.1745-7262.2008.00343.x 18097519

[B20] Han Y. Feng H. L. Sandlow J. I. Haines C. J. (2009). Comparing expression of progesterone and estrogen receptors in testicular tissue from men with obstructive and nonobstructive azoospermia. J. Androl. 30 (2), 127–133. 10.2164/jandrol.108.005157 18835831

[B21] Hess R. A. Fernandes S. A. Gomes G. R. Oliveira C. A. Lazari M. F. Porto C. S. (2011). Estrogen and its receptors in efferent ductules and epididymis. J. Androl. 32 (6), 600–613. 10.2164/jandrol.110.012872 21441425

[B22] Hwang Y. P. Jeong H. G. (2010). Ginsenoside Rb1 protects against 6-hydroxydopamine-induced oxidative stress by increasing heme oxygenase-1 expression through an estrogen receptor-related PI3K/Akt/Nrf2-dependent pathway in human dopaminergic cells. Toxicol. Appl. Pharmacol. 242 (1), 18–28. 10.1016/j.taap.2009.09.009 19781563

[B23] Ishii T. Warabi E. (2019). Mechanism of rapid nuclear Factor-E2-related factor 2 (Nrf2) activation *via* membrane-associated estrogen receptors: Roles of NADPH Oxidase 1, neutral sphingomyelinase 2 and epidermal growth factor receptor (EGFR). Antioxid. (Basel) 8 (3), E69. 10.3390/antiox8030069 PMC6466580 30889865

[B24] Jiang H. Zhu W. J. Li J. Chen Q. J. Liang W. B. Gu Y. Q. (2014). Quantitative histological analysis and ultrastructure of the aging human testis. Int. Urol. Nephrol. 46 (5), 879–885. 10.1007/s11255-013-0610-0 24277275

[B25] Johnson L. Zane R. S. Petty C. S. Neaves W. B. (1984). Quantification of the human Sertoli cell population: its distribution, relation to germ cell numbers, and age-related decline. Biol. Reprod. 31 (4), 785–795. 10.1095/biolreprod31.4.785 6509142

[B26] Johnson L. Thompson D. L. Jr. Varner D. D. (2008). Role of Sertoli cell number and function on regulation of spermatogenesis. Anim. Reprod. Sci. 105 (1-2), 23–51. 10.1016/j.anireprosci.2007.11.029 18242891

[B27] Li D. Meng L. Xu T. Su Y. Liu X. Zhang Z. (2017). RIPK1-RIPK3-MLKL- dependent necrosis promotes the aging of mouse male reproductive system. Elife 6, e27692. 10.7554/eLife.27692 28807105 PMC5557593

[B28] Long M. Yang S. H. Shi W. Li P. Guo Y. Guo J. (2017). Protective effect of proanthocyanidin on mice Sertoli cell apoptosis induced by zearalenone via the Nrf2/ARE signalling pathway. Environ. Sci. Pollut. Res. Int. 24 (34), 26724–26733. 10.1007/s11356-017-0123-y 28956244

[B29] Lucas T. F. Siu E. R. Esteves C. A. Monteiro H. P. Oliveira C. A. Porto C. S. (2008). 17Beta-Estradiol induces the translocation of the estrogen receptors ESR1 and ESR2 to the cell membrane, MAPK3/1 phosphorylation and proliferation of cultured immature rat Sertoli cells. Biol. Reprod. 78 (1), 101–114. 10.1095/biolreprod.107.063909 17928626

[B30] Lucas T. F. Pimenta M. T. Pisolato R. Lazari M. F. Porto C. S. (2011). 17β-estradiol signaling and regulation of Sertoli cell function. Spermatogenesis 1 (4), 318–324. 10.4161/spmg.1.4.18903 22332115 PMC3271643

[B31] Meng X. Lindahl M. Hyvönen M. E. Parvinen M. de Rooij D. G. Hess M. W. (2000). Regulation of cell fate decision of undifferentiated spermatogonia by GDNF. Science 287 (5457), 1489–1493. 10.1126/science.287.5457.1489 10688798

[B32] Meng X. Sun G. Ye J. Xu H. Wang H. Sun X. (2014a). Notoginsenoside R1-mediated neuroprotection involves estrogen receptor-dependent crosstalk between Akt and ERK1/2 pathways: A novel mechanism of Nrf2/ARE signaling activation. Free. Radic. Res. 48 (4), 445–460. 10.3109/10715762.2014.885117 24437944

[B33] Meng X. Wang M. Sun G. Ye J. Zhou Y. Dong X. (2014b). Attenuation of Aβ25-35-induced parallel autophagic and apoptotic cell death by gypenoside XVII through the estrogen receptor-dependent activation of Nrf2/ARE pathways. Toxicol. Appl. Pharmacol. 279 (1), 63–75. 10.1016/j.taap.2014.03.026 24726523

[B34] Miller C. J. Gounder S. S. Kannan S. Goutam K. Muthusamy V. R. Firpo M. A. (2012). Disruption of Nrf2/ARE signaling impairs antioxidant mechanisms and promotes cell degradation pathways in aged skeletal muscle. Biochim. Biophys. Acta 1822 (6), 1038–1050. 10.1016/j.bbadis.2012.02.007 22366763

[B35] Mok S. K. Chen W. F. Lai W. P. Leung P. C. Wang X. L. Yao X. S. (2010). Icariin protects against bone loss induced by oestrogen deficiency and activates oestrogen receptor-dependent osteoblastic functions in UMR 106 cells. Br. J. Pharmacol. 159 (4), 939–949. 10.1111/j.1476-5381.2009.00593.x 20128811 PMC2829219

[B36] Nan Y. Zhang X. Yang G. Xie J. Lu Z. Wang W. (2014). Icariin stimulates the proliferation of rat Sertoli cells in an ERK1/2-dependent manner *in vitro*. Andrologia 46 (1), 9–16. 10.1111/and.12035 23134192

[B37] Naughton C. K. Jain S. Strickland A. M. Gupta A. Milbrandt J. (2006). Glial cell-line derived neurotrophic factor-mediated RET signaling regulates spermatogonial stem cell fate. Biol. Reprod. 74 (2), 314–321. 10.1095/biolreprod.105.047365 16237148

[B38] Oh J. H. Choi M. S. Park H. J. Park S. M. Lee E. H. Kang S. J. (2010). Gene expression profiles of TM4 mouse Sertoli cells after 1,3-dinitrobenzene exposure and analysis of genes related to tight junction signaling pathways. Biochip. J. 4 (4), 313–321. 10.1007/s13206-010-4408-1

[B39] Oliveira C. A. Carnes K. França L. R. Hess R. A. (2001). Infertility and testicular atrophy in the antiestrogen-treated adult male rat. Biol. Reprod. 65 (3), 913–920. 10.1095/biolreprod65.3.913 11514358

[B40] Paniagua R. Amat P. Nistal M. Martin A. (1985). Ultrastructural changes in Sertoli cells in ageing humans. Int. J. Androl. 8 (4), 295–312. 10.1111/j.1365-2605.1985.tb00843.x 2416699

[B41] Petersen P. M. Seierøe K. Pakkenberg B. (2015). The total number of Leydig and Sertoli cells in the testes of men across various age groups - a stereological study. J. Anat. 226 (2), 175–179. 10.1111/joa.12261 25545958 PMC4304573

[B42] Royer C. Lucas T. F. Lazari M. F. Porto C. S. (2012). 17Beta-estradiol signaling and regulation of proliferation and apoptosis of rat Sertoli cells. Biol. Reprod. 86 (4), 108. 10.1095/biolreprod.111.096891 22219213

[B43] Ryu B. Y. Orwig K. E. Oatley J. M. Avarbock M. R. Brinster R. L. (2006). Effects of aging and niche microenvironment on spermatogonial stem cell self-renewal. Stem. Cells 24 (6), 1505–1511. 10.1634/stemcells.2005-0580 16456131 PMC5501308

[B44] Salomon T. B. Hackenhaar F. S. Almeida A. C. Schüller A. K. Gil Alabarse P. V. (2013). Oxidative stress in testis of animals during aging with and without reproductive activity. Exp. Gerontol. 48 (9), 940–946. 10.1016/j.exger.2013.06.010 23834967

[B45] Scuto M. C. Mancuso C. Tomasello B. Ontario M. L. Cavallaro A. Frasca F. (2019). Curcumin, Hormesis and the Nervous System. Nutrients 11 (10), E2417. 10.3390/nu11102417 31658697 PMC6835324

[B46] Sinkevicius K. W. Laine M. Lotan T. L. Woloszyn K. Richburg J. H. Greene G. L. (2009). Estrogen-dependent and -independent estrogen receptor -alpha signaling separately regulate male fertility. Endocrinology 150 (6), 2898–2905. 10.1210/en.2008-1016 19264877 PMC2689797

[B47] Sneddon S. F. Walther N. Saunders P. T. K. (2005). Expression of androgen and estrogen receptors in Sertoli cells: studies using the mouse SK11 cell line. Endocrinology 146 (12), 5304–5312. 10.1210/en.2005-0914 16166216

[B48] Song L. Zhao J. Zhang X. Li H. Zhou Y. (2013). Icariin induces osteoblast proliferation, differentiation and mineralization through estrogen receptor-mediated ERK and JNK signal activation. Eur. J. Pharmacol. 714 (1-3), 15–22. 10.1016/j.ejphar.2013.05.039 23764463

[B49] Tenover J. S. Mclachlan R. I. Dahl K. D. Burger H. G. de Kretser D. M. Bremner W. J. (1988). Decreased seruminhibin levels in normal elderly men: evidence for a decline in Sertoli cell function with aging. J. Clin. Endocrinol. Metab. 67 (3), 455–459. 10.1210/jcem-67-3-455 3137240

[B50] Trovato A. Siracusa R. Di Paola R. Scuto M. Ontario M. L. Bua O. (2016). Redox modulation of cellular stress response and lipoxin A4 expression by Hericium Erinaceus in rat brain: relevance to Alzheimer’s disease pathogenesis. Immun. Ageing. 13, 23. 10.1186/s12979-016-0078-8 27398086 PMC4938991

[B51] Trovato A. Pennisi M. Di Paola R. Scuto M. Crupi R. Cambria M. T. (2018). Neuroinflammation and neurohormesis in the pathogenesis of Alzheimer’s disease and Alzheimer-linked pathologies: modulation by nutritional mushrooms. Immun. Ageing. 15, 8. 10.1186/s12979-017-0108-1 29456585 PMC5813410

[B52] Vogl A. W. Lyon K. Adams A. Piva M. Nassour V. (2018). The endoplasmic reticulum, calcium signaling and junction turnover in Sertoli cells. Reproduction 155 (2), R93–104. 10.1530/REP-17-0281 29066527

[B53] Wang Y. Chen F. Ye L. Zirkin B. Chen H. (2017). Steroidogenesis in Leydig cells: effects of aging and environmental factors. Reproduction 154 (4), R111–R122. 10.1530/REP-17-0064 28747539 PMC5731485

[B54] Wang Y. Guo S. H. Shang X. J. Yu L. S. Zhu J. W. Zhao A. (2018). Triptolide induces Sertoli cell apoptosis in mice via ROS/JNK-dependent activation of the mitochondrial pathway and inhibition of Nrf2-mediated antioxidant response. Acta Pharmacol. Sin. 39 (2), 311–327. 10.1038/aps.2017.95 28905938 PMC5800476

[B55] Weinbauer G. F. Niehaus M. Nieschlag E. (2004). “The role of testosterone in spermatogenesis[M] // Testosterone: Action, deficiency, substitution.” in Springer Berlin Heidelberg. 4, 143–168. 10.1007/978-3-642-72185-4_4

[B56] Wu B. Chen Y. Huang J. Ning Y. Bian Q. Shan Y. (2012). Icariin improves cognitive deficits and activates quiescent neural stem cells in aging rats. J. Ethnopharmacol. 142 (3), 746–753. 10.1016/j.jep.2012.05.056 22687254

[B57] Xiong Y. B. Zhou C. H. (1994). The effect of extracts from Herba Epimedii and Semen Cuscutae on the function of male reproduction. Chin. Pharm. J. 29, 89–91. (in Chinese).

[B58] Yang S. H. Yu L. H. Li L. Guo Y. Zhang Y. Long M. (2018). Protective mechanism of sulforaphane on cadmium-induced Sertoli cell injury in mice testis via Nrf2/ARE signaling pathway. Molecules 23 (7), E1774. 10.3390/molecules23071774 30029485 PMC6100605

[B59] Zhang Z. B. Yang Q. T. (2006). The testosterone mimetic properties of icariin. Asian J. Androl. 8 (5), 601–605. 10.1111/j.1745-7262.2006.00197.x 16751992

[B60] Zhang T. Liang X. Shi L. Wang L. Chen J. Kang C. (2013). Estrogen receptor and PI3K/Akt signaling pathway involvement in S-(-)equol-induced activation of Nrf2/ARE in endothelial cells. PloS One 8 (11), e79075. 10.1371/journal.pone.0079075 24260155 PMC3833998

[B61] Zhao H. Ma N. Liu Z. Wang T. Yuan C. He Y. (2019). Protective effect of Wuzi Yanzong recipe on testicular dysfunction through inhibition of germ cell apoptosis in ageing rats via endoplasmic reticulum stress. Andrologia 51 (2), e13181. 10.1111/and.13181 30393883

[B62] Zhou Y. Chen Y. Hu X. Guo J. Shi H. Yu G. (2019). Icariin attenuate microcystin-LR-induced gap junction injury in Sertoli cells through suppression of Akt pathways. Environ. Pollut. 251, 328–337. 10.1016/j.envpol.2019.04.114 31091496

[B63] Zhu C. Wang S. Wang B. Du F. Hu C. Li H. (2015). 17β-Estradiol up-regulates Nrf2 via PI3K/AKT and estrogen receptor signaling pathways to suppress light-induced degeneration in rat retina. Neuroscience 304, 328–339. 10.1016/j.neuroscience.2015.07.057 26211446

