# Supplementary figures and images for: Icariin Improves Age-Related Testicular Dysfunction by Alleviating Sertoli Cell Injury via Upregulation of the ERα/Nrf2-Signaling Pathway
Source: Front Pharmacol. 2020 May 12;11:677. doi: 10.3389/fphar.2020.00677 (PMC7247842; doi:10.3389/fphar.2020.00677)

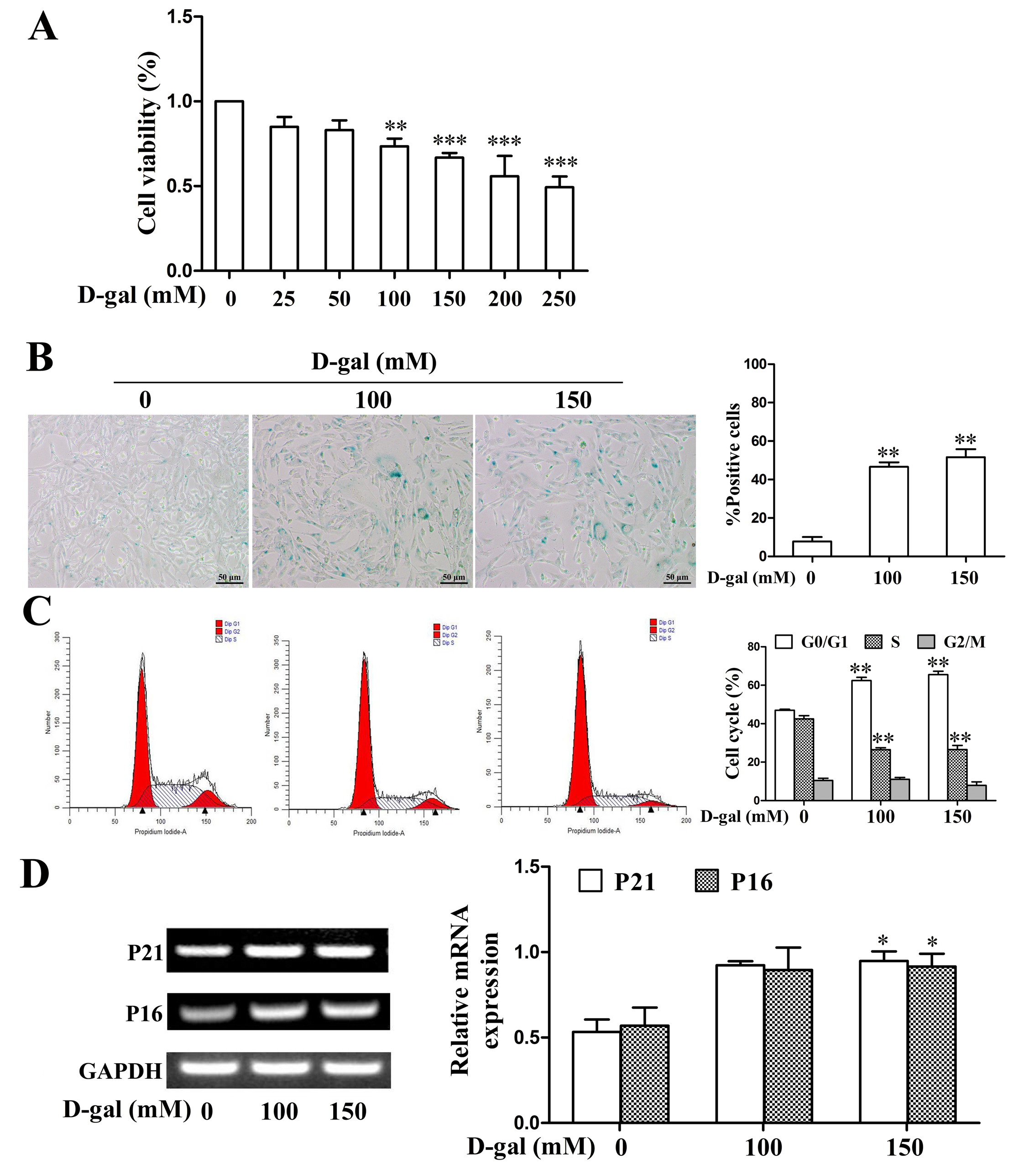

Supplement: Supplementary Figure S1 — D-galactose (D-gal) treatment is sufficient to induce TM4 cell senescence. (A) Cellular viability was detected using MTT assay. TM4 cells at a concentration of 3 × 10^4^ cells /well in 96-well plates were treated with D-gal at various concentrations (0–250 mM) for 60 h. (B–D) TM4 cells at 5 × 10^5^/well in 6-well plates were treated with 100 or 150 mM of D-gal for 60 h. (B) Representative images from SA-β-gal staining of cells (left; scale bar = 50 μm) and the percentages of SA-β-gal-positive cells from a total of 500 cells (right). (C) The cell cycle was detected by flow cytometry. (D) The relative mRNA expression levels of P21 and P16 were measured with RT-PCR. Data are presented as means ± SEM of three independent experiments. *P < 0.05, **P < 0.01 versus control. [file Image_1.tif]

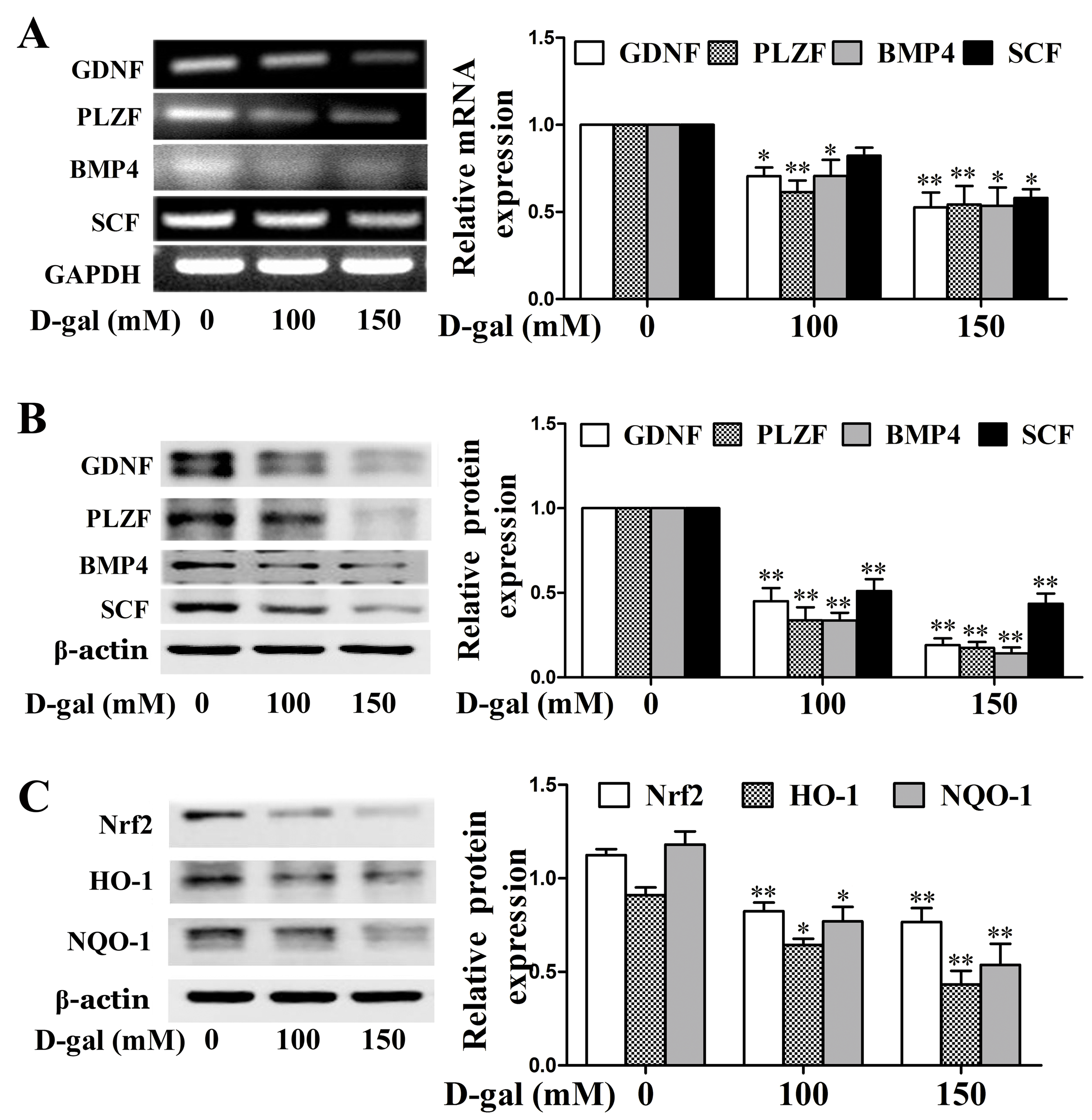

Supplement: Supplementary Figure S2 — D-galactose (D-gal) treatment is effective in inducing a decline in TM4 cell function and Nrf2 signaling. TM4 cells at 5 × 10^5^/well in 6-well plates were treated with 100 or 150 mM of D-gal for 60 h. (A) The relative mRNA expression levels of GDNF, PLZF, BMP4, and SCF were measured with RT-PCR. (B) The relative protein expression levels of GDNF, PLZF, BMP4, and SCF were measured using western immunoblotting analysis. (D) The relative protein expression levels of Nrf2, HO-1 and NQO-1 were measured using western blotting analysis. Data are presented as means ± SEM of three independent experiments. *P < 0.05, **P < 0.01 versus control. [file Image_2.tif]

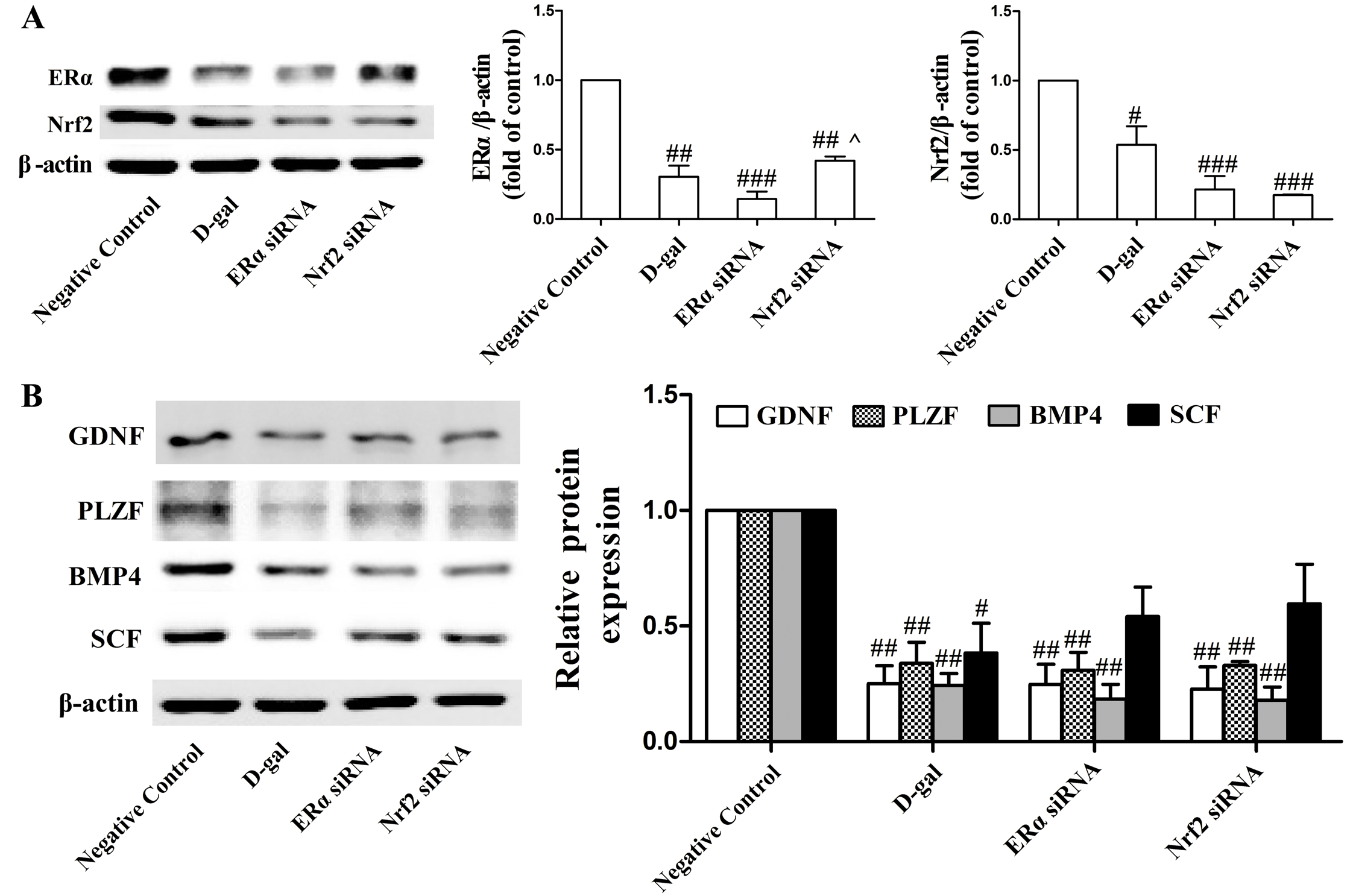

Supplement: Supplementary Figure S3 — Nrf2 is involved in the maintenance of Sertoli cell function. TM4 cells at 1 × 10^5^/well in 6-well plates were treated with D-gal (100 mM) for 60 h or transferred with ERα siRNA for 60 h or Nrf2 siRNA for 72 h. (A) The relative protein expression levels of ERα and Nrf2 in TM4 cells were measured by western blotting analysis. (B) The relative protein expression levels of ERα, GDNF, PLZF, BMP4, and SCF in TM4 cells were measured using western blotting analysis. #P < 0.05, ##P < 0.01, ###P < 0.01 versus negative control; ^P < 0.05, ^^P < 0.01 versus ERα siRNA group. [file Image_3.tif]
