# Supplementary material for: Icariin Improves Age-Related Testicular Dysfunction by Alleviating Sertoli Cell Injury via Upregulation of the ERα/Nrf2-Signaling Pathway
Source: Front Pharmacol. 2020 May 12;11:677. doi: 10.3389/fphar.2020.00677 (PMC7247842; doi:10.3389/fphar.2020.00677)
Supplement: Suplementary Table S1 — Antibodies used in this study. [file Table_1.doc]

**Table 1 Antibodies used in this study**

| Antibody | Corporation | Catalog number | Concentration（IF） | Concentration（WB） |
| --- | --- | --- | --- | --- |
| SOX9 | millipore | AB5535 | 1:200 |  |
| GDNF | abcam | ab18956 |  | 1:500 |
| PLZF | Santa Cruz | sc-28319 |  | 1:500 |
| BMP4 | abcam | ab39973 |  | 1:1000 |
| SCF | Santa Cruz | sc-13126 |  | 1:1000 |
| ERα | Santa Cruz | sc-8005 |  | 1:1000 |
| ERα | Absin | abs131646 | 1:250 |  |
| ERβ | Santa Cruz | sc-390243 |  | 1:1000 |
| Nrf2 | abcam | ab137550 | 1:250 (in vivo) | 1:1000 |
| Nrf2 | abcam | ab31163 | 1:400 (in vitro) |  |
| HO-1 | Santa Cruz | sc-10789 |  | 1:1000 |
| NQO-1 | Servicebio | GB11282 |  | 1:1000 |
| β-actin | Cell Signaling Technology | #4970 |  | 1:5000 |
| Alexa Fluor 594 Donkey Anti-Rabbit lgG (H+L) | Jackson ImmunoResearch | 140422 | 1:200 |  |
| Alexa Fluor488 Donkey Anti-Rabbit (H+L) | Jackson ImmunoResearch | 127725 | 1:500 |  |
